# Recent trends in cutaneous malignant melanoma in the Yorkshire region of England; incidence, mortality and survival in relation to stage of disease, 1993–2003

**DOI:** 10.1038/sj.bjc.6603216

**Published:** 2006-06-06

**Authors:** A Downing, J A Newton-Bishop, D Forman

**Affiliations:** 1 Centre for Epidemiology and Biostatistics, University of Leeds, Leeds, UK; 2 Cancer Research UK Clinical Centre, St James's Hospital, Leeds, UK; 3 Northern and Yorkshire Cancer Registry and Information Service, Arthington House, Cookridge Hospital, Leeds, UK

**Keywords:** malignant melanoma, incidence, mortality, survival

## Abstract

The aim of this study was to investigate recent trends in incidence, mortality and survival in patients diagnosed with malignant melanoma (MM) in relation to stage (Breslow thickness). Cases of primary invasive and *in situ* MM diagnosed between 1st January 1993 and 31st December 2003 in the former Yorkshire Health Authority were identified from cancer registry data. Over the study period, the incidence of invasive MM increased from 5.4 to 9.7 per 100 000 in male subjects and from 7.5 to 13.1 per 100 000 in female subjects. Most of this increase was seen in thin tumours (<1.5 mm). Thin tumours were more likely to be diagnosed in the younger age groups and be classified as superficial spreading melanoma. *In situ* melanoma rates increased only slightly. Over the same time period, mortality rates have been relatively constant in both male and female subjects. Five-year relative survival varied from 91.8% (95% CI 90.4–93.1) for patients with thin tumours to 41.5% (95% CI 36.7–46.3) for those with thick tumours. In multivariable analyses, Breslow thickness was the most important prognostic factor. Age, sex and level of deprivation were also identified as independent prognostic factors. The trends in incidence suggest that the increase is real, rather than an artefact of increased scrutiny, implying that primary prevention in the Yorkshire area of the UK has failed to control trends in incidence. Mortality, in contrast, appears to be levelling off, indicating that secondary prevention has been more effective.

Although time trends in the incidence of and mortality from malignant melanoma (MM) in the UK have been reported by many studies, relatively few studies have looked at these changes in relation to the stage of disease (Melia *et al*, 1995; Mackie *et al*, 1997, 2002; Holme *et al*, 2001). Since the 1990s, the Northern and Yorkshire Cancer Registry and Information Service (NYCRIS) have collected information on Breslow thickness, the most commonly used measure of stage. This has provided us with a unique opportunity to investigate recent trends in the incidence of MM in relation to stage using population-based registry data. Because death certificates do not contain any information on stage of disease, it is not possible to look at equivalent mortality data. By analysing the pattern of survival in the incident cases, it is possible, however, to investigate the effect of changes in the pattern of melanoma incidence on disease outcome.

## DATA AND METHODS

Cases of primary invasive and *in situ* MM (ICD10 (World Health Organization, 1992) codes C43 and D03) diagnosed between 1st January 1993 and 31st December 2003 in the former Yorkshire Health Authority were identified from the NYCRIS database. Relevant patient and tumour characteristics (such as age, sex, Breslow thickness, histological type and body site) were extracted. Breslow thickness was divided into three groups representing thin (<1.5 mm), intermediate (1.5–4.0 mm) and thick (>4.0 mm) tumours, according to the groupings specified on the histopathology forms. As a measure of socioeconomic status, the income domain score of Index of Multiple Deprivation 2000 (Department of the Environment, Transport and the Regions, 2000) was matched with the patient postcode and divided into quintiles.

Incidence rates were calculated as 3-year rolling averages using the 1998 Yorkshire population as a denominator and standardised to the European population. Melanoma mortality rates were obtained from the Cancer Information System (CIS) maintained by NYCRIS (Northern & Yorkshire Cancer Registry & Information Service, 2005). Information on mortality was not available for *in situ* melanoma, therefore the rates presented refer to invasive MM only.

Survival analyses were performed with the survival period defined as the time difference between the date of diagnosis and 5-year follow-up, the date of death or the date of censoring (31st May 2005). *In situ* tumours were excluded from these analyses, as survival rates are very high (∼100%) (American Joint Committee on Cancer, 2002). Five-year relative survival estimates (Ederer *et al*, 1961) were calculated and multivariable analyses were performed using the Cox proportional hazards regression model (Cox, 1972). All analyses were performed using STATA 9.0 (StataCorp, TX, USA).

## RESULTS

### Incidence

Between 1993 and 2003, 5513 patients were diagnosed with MM in the Yorkshire area. Of these, 4178 were diagnosed with invasive disease and 1335 with *in situ* MM. Breslow thickness was recorded in 90.8% of cases overall, increasing from 70.9% in 1993 to 96.2% in 2003.

**Figure 1:**
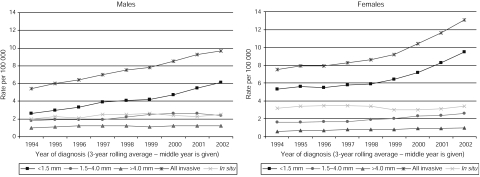
Age standardised incidence rates of MM by Breslow thickness: male and female subjects, Yorkshire UK, 1993–1995 to 2001–2003.

Over the study period, the age standardised incidence of invasive MM in male subjects steadily increased from 5.4 per 100 000 in 1993–1995 to 9.7 per 100 000 in 2001–2003 (Figure 1). In female subjects, the rate increased from 7.5 per 100 000 in 1993–1995 to 13.1 per 100 000 in 2001–2003, with most of the increase occurring after the period 1998–2000. There were slight increases in the incidence of *in situ* tumours over this time period, rising from 2.0 to 2.5 per 100 000 in male subjects and from 3.2 to 3.4 per 100 000 in female subjects.

**Figure 2:**
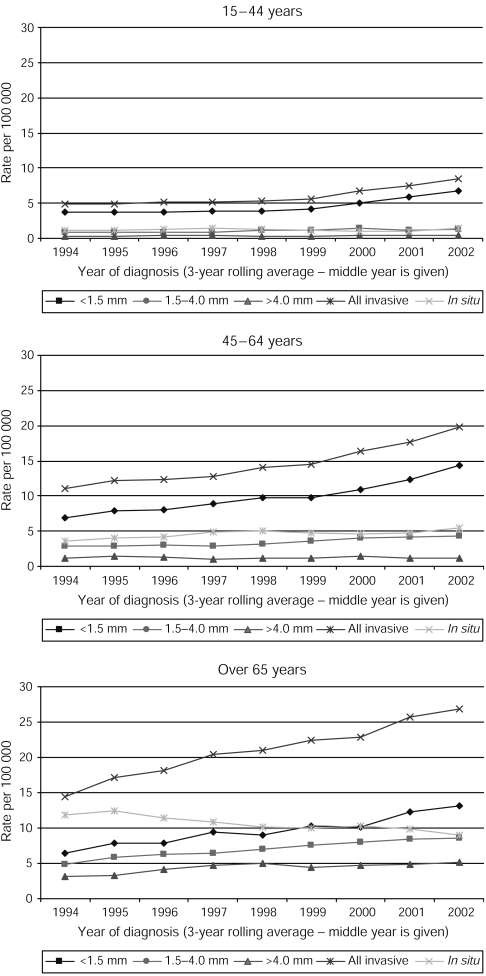
Age standardised incidence rates of MM by Breslow thickness: 15–44 years, 45–64 years, over 65 years, Yorkshire UK, 1993–1995 to 2001–2003.

Looking at the rates by Breslow thickness, the general patterns for invasive MM were mirrored for thin tumours, with increases in all age groups, from 2.6 to 6.1 per 100 000 in male subjects and 5.3 to 9.5 per 100 000 in female subjects during the study period (Figure 1). Proportionally, 77.1% of invasive tumours in the 15- to 44-year age group were classified as thin, compared to 67.8 and 45.2% in those aged 45–64 years and over 65 years. The incidence of intermediate and thick tumours increased slightly in both male and female subjects, with most of the increase in intermediate tumours and all of the increase in thick tumours seen in those aged over 65 years (Figure 2).

**Figure 3:**
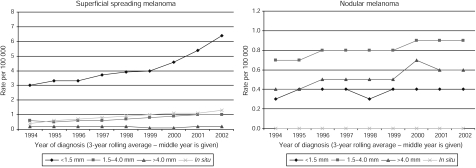
Age standardised incidence rates of malignant melanoma by Breslow thickness: superficial spreading and nodular melanomas, Yorkshire UK, 1993–1995 to 2001–2003.

Superficial spreading melanoma was the most common histological type recorded, followed by nodular melanoma. During the study period, the proportion of cases recorded as superficial spreading melanoma rose from 53 to 64%. The increase in superficial spreading melanoma was driven by an increase in thin tumours, with a slight increase in the incidence of intermediate tumours (Figure 3). For nodular melanoma, the picture was quite different, with the rate of thin tumours being less than that of intermediate and thick tumours. The numbers of acral lentiginous and lentigo maligna melanomas were too small to observe any clear patterns.

Analysis by body site revealed that in male subjects the increases in thin tumours were mainly seen for the trunk. In female subjects, the rates remained much higher for tumours of the lower limb. However, increases were seen in female subjects for all body sites, with a doubling of the rate of thin tumours of the head and neck, trunk and upper limbs (data not shown).

### Mortality

**Figure 4:**
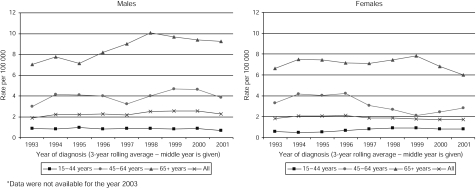
Age standardised mortality rates of invasive cutaneous malignant melanoma by sex, Yorkshire UK, 1993–1995 to 2001–2003. ^*^Data were not available for the year 2003.

There was a steady decrease in the female age standardised mortality rate after the period 1995–1997, from 2.7 per 100 000 to 2.2 per 100 000 in 2000–2002 (Figure 4). The pattern in male subjects was less clear. In 1993–1995, the rate was 2.9 per 100 000 and this remained steady until 1996–1998, after which it increased to a peak of 3.3 per 100 000 in 1998–2000 and then decreased to 2.9 per 100 000 in 2000–2002. Mortality appears to have stabilised or decreased in recent years across all age groups, except for a slight increase in the rate for women aged 45–64 years.

### Survival

Overall 5-year relative survival was 76.8% (95% CI 75.3–78.2). This varied from 70.0% (95% CI 67.4–72.3) for male subjects to 81.5% (95% CI 79.7–83.2) for female subjects. In relation to Breslow thickness, survival was 91.8% (95% CI 90.4–93.1) for patients with thin tumours, 63.5% (95% CI 60.0–66.7) for those with intermediate tumours and 41.5% (95% CI 36.7–46.3) for those with thick tumours.

In multivariable analyses, Breslow thickness was the most important prognostic factor, with a hazard ratio (HR) of 6.06 (95% CI 4.64–7.93) for patients with thick tumours compared to those with thin tumours, after adjustment for age, sex, histological type body site and deprivation quintile (Table 1). Female survival was significantly better than that in male subjects after adjustment (HR=0.67; 95% CI 0.56–0.80) and older age was associated with an increased risk of death (HR=1.04; 95% CI 1.04–1.05 per annum). Patients living in the two most deprived quintiles had a worse prognosis (HR=1.40; 95% CI 1.05–1.88 for quintile 5 compared with quintile 1). There was no change in survival in relation to individual year of diagnosis (HR=1.00; 95% CI 0.97–1.03).

## DISCUSSION

In this population-based cancer registry study, we have shown that, over the 10-year period to 2003, the incidence of invasive MM has increased in both male and female subjects in the Yorkshire region of England. In female subjects, the increase has been particularly pronounced since 1998 and comes after a period of relative stability. These results suggest a failure to stem the rise in incidence of this form of cancer, despite efforts to the contrary.

These trends compare well with those from other studies. Several authors reported a levelling off in incidence rates in the 1990s in the UK (Mackie *et al*, 1997, 2002; Newnham and Moller, 2002) and other white Caucasian populations (Hall *et al*, 1999; Marrett *et al*, 2001; de Vries *et al*, 2003). Recent MM incidence rates for the UK, covering the period 1993–2002, show similar trends, with relatively stable incidence rates between 1993 and 1999 but a sharp rise in incidence thereafter (Cancer Research UK, 2005). There is, therefore, a high degree of conformity in the incidence trends between Yorkshire and the UK overall.

For both sexes, the increases have been predominantly of thin, early-stage tumours, as has been the trend in other populations (Hall *et al*, 1999; Marrett *et al*, 2001; de Vries *et al*, 2004). Increases in thick tumours were seen mainly in the older age groups and similar trends have been observed elsewhere (Murray *et al*, 2005). The observed increase in thin tumours may suggest a trend towards earlier diagnosis due to increased awareness, but public health campaigns directed towards early detection have been in existence since 1987 (Melia *et al*, 1995) and so this does not fully explain the increase in female subjects seen after 1999. During the 1980s and the 1990s, as a part of these public health campaigns, a number of pigmented lesion clinics were opened. These may have added to the increase in the diagnosis of thin tumours, while as yet having little effect on thicker tumours. In the Yorkshire region, we are aware of a number of such clinics but when the data are broken down by Cancer Network (data not shown), the observed variation does not correspond with the location of these clinics. It seems unlikely, therefore, that the opening of these clinics explains the overall increased incidence.

The proportion of tumours classified as superficial spreading melanomas (predominantly thin tumours) increased in the time period studied as has been reported previously in Scotland (MacKie *et al*, 2002). An increasing incidence of MM, particularly of thinner tumours, has led some authors to suggest that these trends are at least in part artefactual. Welch *et al* (2005) have suggested that increases in MM seen in the US could represent an ‘overdiagnosis’ owing to increased scrutiny and an increased number of biopsies being taken. They reported a correlation between MM rates and biopsy rates in those aged over 65 years, whereas mortality rates remained stable. In response, de Vries and Coebergh (2005) note that in many European countries mortality from MM has continued to increase, especially in older men, suggesting that part of the observed increases are real. Furthermore, despite the increases seen in thin tumours in our data, the incidence of *in situ* tumours has remained relatively constant. If the reported increase in the incidence of thin tumours was a function of increased biopsy rates, then we would have expected a similar, or even larger, increase in *in situ* lesions. Finally, whereas a large proportion of the increase in incidence has been seen in thin tumours, the incidence of intermediate and thick tumours has increased slightly, whereas we would expect these to begin to decrease if it was simply earlier detection (lead-time bias).

This increase in thin tumours may, however, represent a change in the type of melanoma being removed, which may in turn result from an increase in intermittent sun exposure. Superficial spreading melanoma may have a stronger relationship to this form of exposure than other melanomas (de Vries and Coebergh, 2004) and evidence certainly suggests that the popularity of sunny holidays with high levels of sun exposure continues to increase in the UK population (National Statistics, 2005).

Despite the adverse association between tumour thickness and risk of death (Holme *et al*, 2001; MacKie *et al*, 2002) and the increased incidence of thicker tumours in older people, mortality rates in those aged over 65 years do not appear to have increased in the past few years in Yorkshire. Although this study suggests a failure of primary prevention, the levelling off of mortality despite the increased incidence supports the view that secondary prevention (early detection) has been more effective. Some authors have challenged this view (Burton and Armstrong, 1994; Swerlick and Chen, 1996) and have suggested that increased surveillance has led to the removal of superficial spreading melanoma with reduced malignant potential. This possibility will be explored as greater understanding of the biological behaviour of tumours with differing patterns of somatic mutations becomes clearer (Curtin *et al*, 2005).

Breslow thickness remains the most important prognostic factor in survival from MM, as shown in our multivariable analysis. Male subjects have worse survival outcomes than female subjects, even after adjusting for the effects of age, Breslow thickness, histology and body site, although this sex effect is not yet understood. Deprivation also increases mortality from MM and this effect remains after adjustment for other known determinants of survival. This observation has been reported before in studies from North America (Berwick, 1999) and Scotland (Mackie and Hole, 1996). There are no treatments known to affect survival from MM and this, therefore, has important implications for our understanding of human responses to cancer. The presence of ulceration is also known to affect prognosis and this has now been incorporated into the American Joint Committee on Cancer staging system (American Joint Committee on Cancer, 2002). In this study, however, we were unable to include information on ulceration, as this was only available in 27% of cases.

In conclusion, the incidence of MM continues to rise in the Yorkshire region: indeed a marked increase in female subjects has occurred in the period since 1999. This study therefore supports the view that primary prevention in this part of the UK has been inadequate to control the increased incidence seen in the UK since the beginning of the 20th century. Mortality appears to be levelling off, which supports the view that secondary prevention has been more effective. UK populations are travelling increasingly to sunny destinations, leading to increased intermittent sun exposure. This seems likely to have the effect of increasing incidence further in future years, albeit predominantly of good prognosis thin tumours.

## Figures and Tables

**Table 1:** Unadjusted and adjusted Cox proportional hazards regression analysis of 5-year survival of patients with cutaneous malignant melanoma

**Variable**	***N***	**Unadjusted HR (95% CI)**	**Adjusted HR^a^ (95% CI)**
*Age*			
Change per year	3127	1.05 (1.05–1.06)	1.04 (1.04–1.05)
			
*Sex*			
Male	1238	1.00	1.00
Female	1899	0.57 (0.48–0.67)	0.67 (0.56–0.80)
			
*Breslow* b			
<1.5 mm	2012	1.00	1.00
1.5–4.0 mm	757	5.58 (4.49–6.92)	3.62 (2.85–4.60)
>4.0 mm	358	12.23 (9.78–15.31)	6.06 (4.64–7.93)
			
*Site* b			
Head and neck	553	1.00	1.00
Lower limb	1104	0.48 (0.38–0.60)	0.90 (0.70–1.17)
Trunk	832	0.65 (0.52–0.82)	1.16 (0.91–1.50)
Upper limb	638	0.50 (0.39–0.65)	0.91 (0.70–1.20)
			
*Histology* b			
Acral lentiginous	63	1.00	1.00
Lentigo maligna	183	0.64 (0.36–1.14)	0.82 (0.44–1.52)
Nodular	753	1.52 (0.94–2.44)	1.28 (0.78–2.09)
Superficial spreading	2128	0.36 (0.22–0.57)	1.02 (0.62–1.68)
			
*IMD quintile*			
1 (affluent)	1014	1.00	1.00
2	892	1.01 (0.80–1.27)	1.06 (0.84–1.33)
3	475	1.18 (0.91–1.53)	1.08 (0.83–1.41)
4	455	1.65 (1.30–2.11)	1.53 (1.20–1.96)
5 (deprived)	274	1.63 (1.22–2.17)	1.40 (1.05–1.88)
			
*Year*			
Change per year	3127	1.00 (0.97–1.03)	1.00 (0.97–1.03)

HR=hazard ratio; IMD=Index of Multiple Deprivation.

a Each variable is adjusted for all other covariates in the table.

b Where information was missing or recorded as ‘unspecified’, the records have been excluded from the analysis.
